# Fenugreek with reduced bitterness prevents diet-induced metabolic disorders in rats

**DOI:** 10.1186/1476-511X-11-58

**Published:** 2012-05-29

**Authors:** Etsuko Muraki, Hiroshige Chiba, Keiko Taketani, Shohei Hoshino, Nobuaki Tsuge, Nobuyo Tsunoda, Keizo Kasono

**Affiliations:** 1 Department of Clinical Dietetics & Human Nutrition, Faculty of Pharmaceutical Sciences, Josai University, Saitama, 350-0295, Japan; 2 House Foods Corporation, Somatech Center, Chiba, 284-0033, Japan

**Keywords:** FRB, High-fat high-sucrose diet, Diet-induced metabolic disorders, Rats

## Abstract

**Background:**

Various therapeutic effects of fenugreek (*Trigonella foenum-graecum* L.) on metabolic disorders have been reported. However, the bitterness of fenugreek makes it hard for humans to eat sufficient doses of it for achieving therapeutic effects. Fenugreek contains bitter saponins such as protodioscin. Fenugreek with reduced bitterness (FRB) is prepared by treating fenugreek with beta-glucosidase. This study has been undertaken to evaluate the effects of FRB on metabolic disorders in rats.

**Methods:**

Forty Sprague–Dawley rats were fed with high-fat high-sucrose (HFS) diet for 12 week to induce mild glucose and lipid disorders. Afterwards, the rats were divided into 5 groups. In the experiment 1, each group (n = 8) was fed with HFS, or HFS containing 2.4% fenugreek, or HFS containing 1.2%, 2.4% and 4.8% FRB, respectively, for 12 week. In the experiment 2, we examined the effects of lower doses of FRB (0.12%, 0.24% and 1.2%) under the same protocol (n = 7 in each groups).

**Results:**

In the experiment 1, FRB dose-dependently reduced food intake, body weight gain, epididymal white adipose tissue (EWAT) and soleus muscle weight. FRB also lowered plasma and hepatic lipid levels and increased fecal lipid levels, both dose-dependently. The Plasma total cholesterol levels (mmol/L) in the three FRB and Ctrl groups were 1.58 ± 0.09, 1.45 ± 0.05*, 1.29 ± 0.07* and 2.00 ± 0.18, respectively (*; *P* < 0.05 vs. Ctrl). The Hepatic total cholesterol levels (mmol/g liver) were 0.116 ± 0.011, 0.112 ± 0.006, 0.099 ± 0.007* and 0.144 ± 0.012, respectively (*; *P* < 0.05 vs. Ctrl). The calculated homeostasis model assessment as an index of insulin resistance (HOMA-IR) indicated 0.52 ± 0.04*, 0.47 ± 0.06*, 0.45 ± 0.05* and 1.10 ± 0.16, respectively (*; *P* < 0.05 vs. Ctrl). None of the FRB groups showed any adverse effect on the liver, kidney or hematological functions. In the experiment 2, no significant difference of food intake was observed, while the 1.2% FRB group alone showed nearly the same effects on glucose and lipid metabolism as in the experiment 1.

**Conclusions:**

Thus we have demonstrated that FRB (1.2 ~ 4.8%) prevents diet-induced metabolic disorders such as insulin resistance, dyslipidemia and fatty liver.

## Background

Recently, under the rapid economic development in advanced countries, lifestyle diseases including metabolic disorders are expanding. The principle of the basic therapeutic policy for these diseases consists of improvement of diet habits and enhancement of physical activities. However, it is difficult to control and change one’s established life styles. In such cases, functional foods with therapeutic effects on metabolic disorders are very helpful for the improvement of lifestyle diseases.

Various medicinal properties of fenugreek (*Trigonella foenum-graecum* L.) have been described earlier and its use in traditional Indian medicine is well known [1]. In recent years, fenugreek has been used in Western countries as a medicinal herb or as a spice. It has been reported that fenugreek reduced serum cholesterol level [2,3], and improved blood glucose level and lipid metabolism in disease model animals of type 1 diabetes mellitus induced by such drug administration as alloxan [4,5] and streptozotocin [6]. Recently, it has been reported that some active components of fenugreek, 4-hydroxyisoleucine (4-OH-Ile) [7,8] and galactomannan [9,10], inhibited the elevation of blood glucose level and improved lipid metabolism *in vivo*. We have also reported that fenugreek reduced lipid levels in plasma and liver, leading to the improvement of insulin sensitivity in rats with metabolic disorders induced by a high-fat high-sucrose diet [11]. In our recent experiments, fenugreek dose-dependently inhibited the lipid accumulation in liver by increasing the excretion of lipid and bile acids in feces. An effective, safe and tolerable dose of fenugreek was about 2.5% (w/w) [12]. However, the strong bitterness of fenugreek makes it difficult for humans to eat sufficient amount of fenugreek for the improvement of metabolic disorders. The main component of bitterness in fenugreek is protodioscin [13] Additional file 1. So protodioscin was enzymatically converted into dioscin with no bitter taste.

The purpose of this study was to examine whether fenugreek with reduced bitterness (FRB) has such effects on diet-induced metabolic disorders as unmodified fenugreek, and to determine the most effective and tolerable dose of FRB.

## Methods

### Materials

Fenugreek and FRB were provided by House Foods (Chiba, Japan). FRB was made by adding cellulase enzyme (SPEZYME CP; Genencor, CA, USA) to fenugreek [14]. 82% of protodioscin was converted into dioscin in the FRB used for the experiment 1, and 96% of protodioscin was converted into dioscin in the FRB used for the experiment 2. The enzyme also digested insoluble fiber fraction, hemicellulose and cellulose in fenugreek, and increased water-soluble fiber fractions in FRB. And the high molecular weight galactomannan (average molecular weight 2,000,000) in fenugreek was converted to the low molecular weight galactomannan (average molecular weight 500,000) in FRB. Thus FRB has a lower viscosity than fenugreek. As a result, FRB contains a larger amount of dioscin and soluble fiber fractions as compared with fenugreek itself.

### Animal care

Three-week-old male, Sprague–Dawley rats were obtained from CLEA Japan, Inc. (Tokyo, Japan). The rats were housed individually in stainless steel wire-bottom cages in a room maintained at 22 ± 2°C and 55 ± 5% relative humidity with a 12 h cycle of light and dark. Tap water was given them freely throughout the experiment. The rats were fed a commercial diet (CE-2; CLEA Japan) for 1 week before proceeding to diet-induced metabolic disorders. Diets used in this study were high-fat high-sucrose (HFS; lard 50%kcal, sucrose 25%kcal) diets based on the modified version of the AIN-93 G [15]. The rats were fed high-fat high-glucose (HFS) diet ad libitum for 12 week to induce metabolic disorders. In both experiments 1 and 2, the rats with diet-induced metabolic disorders were randomly divided into 5 groups of 7–8 rats each. Each experimental group was fed with either of the experimental diets shown in Table 1 for an additional 12 week period. After the 12 week, the rats were individually transferred into metabolic cages, and their feces were separately collected for 1 day. At the end of the experiment, the rats were brought to rest under pentobarbital sodium (100 mg/kg, ip) anesthesia. Their blood samples were collected from the abdominal aorta, and the plasma was separated by means of centrifugation (2,000 × g for 20 min at 4°C) and stored at −30°C until analyzed. Immediately after the collection of the blood samples, the livers, the solei and the epididymal white adipose tissues (EWAT) were excised, weighed and stored at −30°C for further analyses.

**Table 1:** **The diets**^**1**^**of experiment 1 and 2**

**(g)**	**Ctrl**	**Fen**	**FRB**				
			**0.12%**	**0.24%**	**1.2%**	**2.4%**	**4.8%**
Lard^2^	260.000	258.626	259.912	259.823	259.115	258.231	256.461
Milk casein^2^	200.000	193.205	199.653	199.306	196.529	193.059	186.118
Sucrose^2^	312.000	312.000	312.000	312.000	312.000	312.000	312.000
α-Corn starch^2^	32.487	32.487	32.487	32.487	32.487	32.487	32.487
β-Corn starch^2^	97.461	92.345	97.050	96.638	93.347	89.232	81.004
Vitamin mixture^3^	10.000	10.000	10.000	10.000	10.000	10.000	10.000
Mineral mixture^4^	35.000	34.264	34.953	34.907	34.535	34.070	33.139
Cellulose powder^2^	50.000	40.045	49.695	49.389	46.947	43.893	37.787
L-Cystin^5^	3.000	3.000	3.000	3.000	3.000	3.000	3.000
t-Butylhydroquinone^5^	0.052	0.052	0.052	0.052	0.052	0.052	0.052
Fenugreek^6^	-	23.976	-	-	-	-	-
FRB^7^	-	-	1.199	2.398	11.988	23.976	47.952

Ctrl: Control group, Fen: Fenugreek group. ^1^Based on the AIN-93 G and modified. ^2,3,4^Purchased from Oriental Yeast Co.,Tokyo, Japan. ^3^Vitamin mixture was based on the AIN-93 formation, and added with tartaric acid choline. ^4^Mineral mixture was based on the AIN-93 G formation. ^5^Purchased from Wako Pure Chemical Industries, Osaka, Japan. ^6,7^Purchased from House Foods Corp., Somatech Center, Chiba, Japan. ^7^FRB: Fenugreek with reduced bitterness.

The experimental protocol was approved by the Institutional Animal Care and Use Committee of Josai University.

### Experimental diets

The experimental diets of the experiment 1: HFS (Ctrl group) as control, the HFS containing 2.4% fenugreek (Fen group), 1.2, 2.4 and 4.8% FRB (Table 1).

The experimental diets of the experiment 2: HFS (Ctrl group) as control, the HFS containing 2.4% fenugreek (Fen group), 0.12, 0.24 and 1.2% FRB.

Water-soluble dietary fiber (SDF) and insoluble dietary fiber (IDF) were measured by the Modified Prosky Method [16]. Fenugreek contains 9.4% (w/w) of SDF and 32.1% (w/w) of IDF, whereas FRB in the experiment 1 and 2 contained 13.4% (w/w) of SDF and 12.6% (w/w) of IDF, and 20.8% (w/w) of SDF and 12.7% (w/w) of IDF, respectively.

### Oral glucose tolerance tests and intraperitoneal insulin tolerance tests

At the10th wk, the rats were deprived of food for 12 h, and afterwards they were submitted to an oral glucose tolerance test (OGTT: 2 g/kg b.w. glucose). And at the 11th wk, an intraperitoneal insulin tolerance test (IPITT: 0.75 U/kg b.w. human regular insulin) was performed. We measured blood glucose concentrations by Ascensia^TM^ DexterZII (Bayer Medical, Tokyo, Japan) using tail blood samples at 0, 30, 60 and 120 min after the glucose administration, and at 0, 15, 30, 60 and 120 min after the insulin administration. Likewise, we measured plasma insulin concentrations by commercial kit (Rat Insulin ELISA KIT, Shibayagi, Co., Gunma, Japan) using separated tail plasma samples at 0, 30, 60 and 120 min after the glucose administration.

### Blood analysis

Plasma triglyceride, total cholesterol, AST and ALT were measured by colorimetric slides using the Fuji Dri-Chem 3500 (Fujifilm Corp., Tokyo, Japan).

### Hepatic and fecal lipid concentrations

The hepatic and fecal lipids were extracted in accordance with the method of Bligh and Dyer [17] and the method of Folch [18], respectively. Each extract was solubilized by Triton-X100 (Wako Pure Chemical Industries, Osaka, Japan), and the lipid concentrations were determined enzymatically using commercial kits (Triglycerides E-Test Wako, Cholesterol E-Test Wako, Total Bile Acid Test Wako; Wako Pure Chemical Industries).

### Statistical analysis

All data are expressed as the mean ± SEM. Statistical analyses were carried out by means of the Statistical Package for Social Sciences (SPSS12.0 J for Windows; SPSS Japan Inc., Tokyo, Japan). The effects of treatment were analyzed by the methods as follows. In the five-group comparison, the variances were calculated by Levene's test. When the variances were unequal among the groups, statistical comparisons were made by a nonparametric test, Kruskal-Wallis test, using post-hoc Mann–Whitney U tests with the Bonferroni correction. When the variances were equal among the groups, statistical comparisons were made by a parametric test, one-way ANOVA, using post-hoc Tukey HSD test. In the two-group comparison, the variances were calculated by Levene's test and statistical comparisons were made with *T*-test. In OGTT and IPITT, statistical comparisons were made by means of repeated ANOVA, using post-hoc Tukey HSD test. Differences were considered significant at *P* < 0.05.

## Results

### Experiment 1

**Figure 1:**
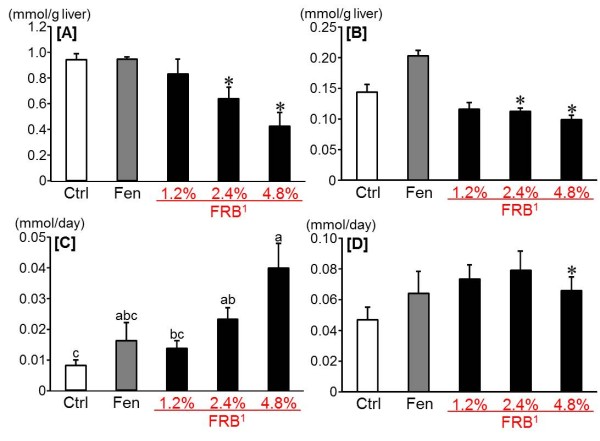
**Effect of FRB on lipids in experiment 1.** Hepatic triglyceride [A] and total cholesterol [B] levels. Fecal triglyceride [C] and total cholesterol [D] levels. Ctrl: Control group, Fen: Fenugreek group. ^1^FRB contains 82% of protodioscin converted to dioscin. Values are expressed as Mean ± SEM for 8 rats. Mean without a common superscript letter is significantly different, *P* < 0.05 (ANOVA). Asterisks indicate a difference from Ctrl: *P* < 0.05 (*t*-test)

**Figure 2:**
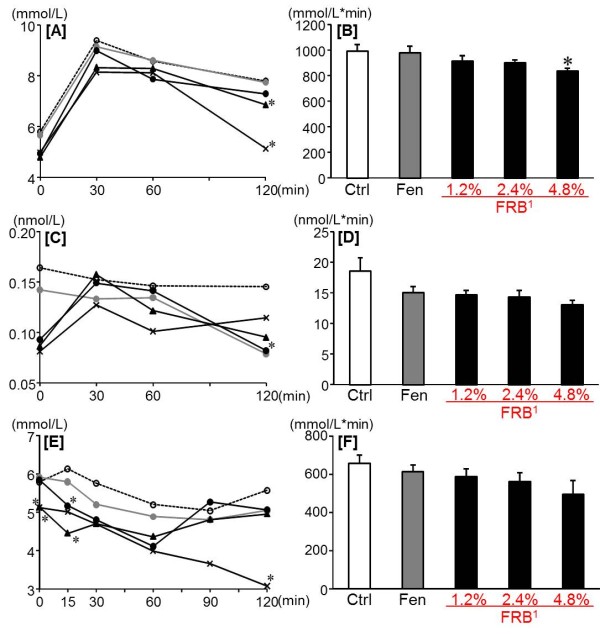
**Effect of FRB on blood insulin and glucose levels in OGTT and IPITT in experiment 1.** Changes of blood glucose [A], AUC of blood glucose [B], change of plasma insulin [C] and AUC of plasma insulin [D] after oral glucose load (2 g/kg). Change of blood glucose [E] and AUC of blood glucose [F] after intraperitoneal insulin load (0.75 U/kg). Ctrl: Control group (), Fen: Fenugreek group (), 1.2% FRB group (), 2.4% FRB group (), 4.8% FRB group ().^1^FRB contains 82% of protodioscin converted to dioscin. Values are expressed as mean for 8 rats. Asterisks indicate a difference from Ctrl: *P* < 0.05 (*t*-test). [A] shows a significant difference in the 4.8% FRB group for the Ctrl group, and [C] shows a significant difference in the 2.4% FRB group for the Ctrl group: *P* < 0.05 (repeated ANOVA).

After 12 week of dietary treatment, the final body weight, the liver weight, the EWAT weight, the soleus muscle weight and the energy efficiency ratio (EER) were reduced by FRB dose-dependently (Table 2). Likewise, the plasma total cholesterol and the hepatic total cholesterol and triglyceride were reduced by FRB dose-dependently, whereas no significant difference was found in the plasma triglyceride (Figure 1A-D). Futhermore, the fasting blood glucose and plasma insulin levels decreased significantly in all FRB groups as compared with the Ctrl and Fen groups. Therefore, homeostasis model assessment as an index of insulin resistance (HOMA-IR) decreased significantly in all FRB groups as compared with the Ctrl and Fen groups (Table 3). And in the 4.8% FRB group, the area under the curve (AUC) of glucose in OGTT was significantly reduced as compared with the Ctrl group (Figure 2B). Moreover, the plasma glucose level decreased remarkably in the 4.8% FRB group at 120 min in OGTT and IPITT as compared with the other groups (Figure 2A,C).

**Table 2:** Effect of fenugreek on the body weight, energy intake, tissue weights and plasma parameters of experiment 1

	**Ctrl**			**Fen**			**FRB**^**6**^								
							**1.2%**			**2.4%**			**4.8%**		
Total energy intake (kJ)	32,156	±	225^a^	32,233	±	175^a^	32,017	±	177^a^	28,789	±	377^b^	26,268	±	1,240^b^
Final body weight (g)	754	±	22^a^	748	±	16^a^	714	±	9^a^	625	±	16^b^	546	±	19^c^
EER^1^ (mg/kJ)	3.95	±	0.37^a^	4.05	±	0.46^a^	2.75	±	0.14^a^	0.80	±	0.23^b^	−2.98	±	0.58^c^
Liver weight (g)	24.2	±	0.9^a^	23.2	±	1.3^a^	21.5	±	0.8^ab^	18.9	±	0.5^bc^	16.6	±	0.5^c^
EWAT^2^ weight (g)	23.7	±	1.3^a^	23.8	±	1.1^a^	21.4	±	1.2^a^	12.8	±	1.2^b^	7.9	±	1.0^c^
Soleuse weight (g)	0.19	±	0.01^ab^	0.21	±	0.01^a^	0.15	±	0.02^bc^	0.13	±	0.01^c^	0.11	±	0.01^c^
Plasma TG^3^ (mmol/L)	2.67	±	0.53	3.05	±	0.57	4.03	±	0.64	3.46	±	0.61	2.93	±	0.52
Plasma TC^4^ (mmol/L)	2.00	±	0.18	1.84	±	0.15	1.58	±	0.09	1.45	±	0.05*	1.29	±	0.07*
AST (U/L)	65.3	±	5.4	53.1	±	2.6	52.3	±	2.6*	53.3	±	1.4*	56.0	±	3.2
ALT (U/L)	22.5	±	3.3	18.9	±	1.3	29.6	±	2.2*	40.1	±	1.7*	51.8	±	3.7*
AST/ALT^5^	1.13	±	0.09^a^	1.05	±	0.09^a^	0.65	±	0.02^bc^	0.49	±	0.02^c^	0.04	±	0.03^c^

Ctrl: Control group, Fen: Fenugreek group. ^1^EER (mg/kJ) = Body weight gain (mg)/Total food intake (kJ). ^2^EWAT: Epididymal white adipose tissue. ^3^TG: Triglyceride. ^4^TC: Total cholesterol. ^5^The relative values for Normal rats' data; AST/ALT = AST (U/L)/ALT (U/L)/Normal rats' data. ^6^FRB contains 82% of protodioscin converted to dioscin. Values are expressed as Mean ± SEM for 8 rats. Mean without a common superscript letter is significantly different, *P* < 0.05 (ANOVA). Asterisks indicate a difference from Ctrl: *P* < 0.05 (*T*-test).

**Table 3:** The insulin resistance index of experiment 1

	**Ctrl**			**Fen**			**FRB**^**4**^								
							**1.2%**			**2.4%**			**4.8%**		
FBG^1^ (mmol/L)	5.79	±	0.29	5.66	±	0.25	4.93	±	0.18*	4.78	±	0.19*	4.98	±	0.23*
FPI^2^ (nmol/L)	0.164	±	0.024^a^	0.142	±	0.009^b^	0.093	±	0.008^ac^	0.086	±	0.010^ac^	0.081	±	0.010^c^
HOMA-IR^3^	1.10	±	0.16^a^	0.93	±	0.08^a^	0.52	±	0.04^b^	0.47	±	0.06^b^	0.45	±	0.05^b^

Ctrl: Control group, Fen: Fenugreek group. ^1^FBG: Fasting blood glucose. ^2^FPI: Fasting plasma insulin. ^3^Relative values of (fasting blood glucose × fasting blood insulin) to the mean value of Ctrl group. ^4^FRB contains 82% of protodioscin converted to dioscin. Values are expressed as Mean ± SEM for 8 rats. Mean without a common superscript letter is significantly different, *P* < 0.05 (ANOVA). Asterisks indicate a difference from Ctrl: *P* < 0.05 (*T*-test).

### Experiment 2

**Figure 3:**
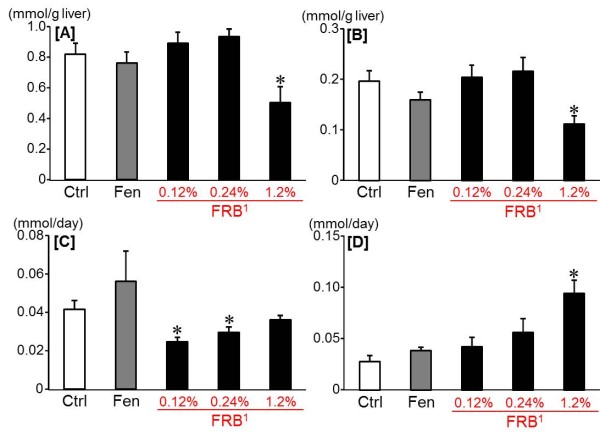
**Effect of FRB on lipids in experiment 2.** Hepatic triglyceride [A] and total cholesterol [B] levels. Fecal triglyceride [C] and total cholesterol [D] levels. Ctrl: Control group, Fen: Fenugreek group. ^1^FRB contains 96% of protodioscin converted to dioscin. Values are expressed as Mean ± SEM for 7–8 rats. Mean without a common superscript letter is significantly different, *P* < 0.05 (ANOVA). Asterisks indicate a difference from Ctrl: *P* < 0.05 (*t*-test)

**Figure 4:**
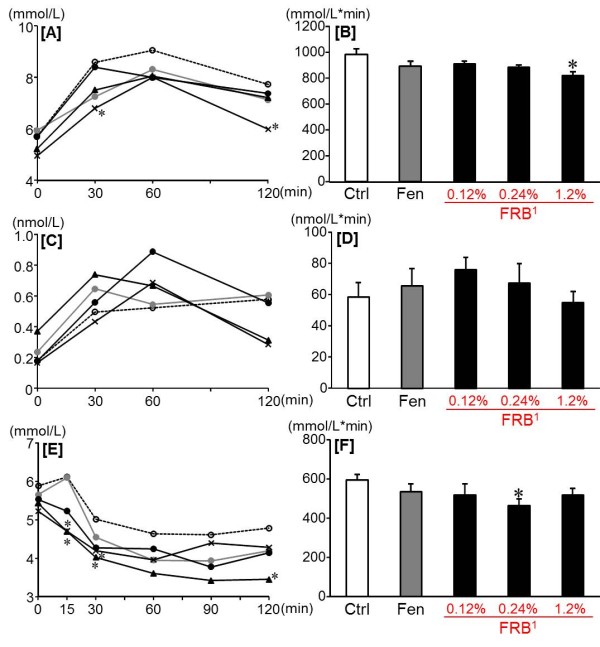
**Effect of FRB on blood insulin and glucose levels in OGTT and IPITT in experiment 2.** Changes of blood glucose [A], AUC of blood glucose [B], change of plasma insulin [C] and AUC of plasma insulin [D] after oral glucose load (2 g/kg). Change of blood glucose [E] and AUC of blood glucose [F] after intraperitoneal insulin load (0.75 U/kg). Ctrl: Control group (), Fen: Fenugreek group (), 0.12% FRB group (), 0.24% FRB group (), 1.2% FRB group (). ^1^FRB contains 96% of protodioscin converted to dioscin. Values are expressed as mean for 7–8 rats. Asterisks indicate a difference from Ctrl: *P* < 0.05 (*t*-test).

After 12 week of dietary treatment, no significant differences were found in body weight gain between the FRB groups and the Ctrl group, whereas the EER, the EWAT weight, the plasma total cholesterol and the hepatic total cholesterol and triglyceride decreased significantly only in the 1.2% FRB group in comparison with the Ctrl group (Table 4 and Figure 3B-D). And the soleus muscle weight and the fecal total cholesterol increased significantly in the 1.2% FRB group as compared with the Ctrl and Fen groups (Table 4, Figure 3E,F). Futhermore, the fasting blood glucose levels decreased significantly in the 1.2% FRB group as compared with the Ctrl group (Table 5). And the AUC of glucose in the OGTT significantly decreased in the 1.2% FRB group as compared with the Ctrl group, whereas the AUC of glucose in the IPTT significantly decreased in the 0.24% FRB group as compared with the Ctrl group (Figure 4B, F). Moreover, in the OGTT, the plasma glucose levels in the 1.2% FRB group were significantly lower at 30 and 120 min than those in the Ctrl group (Figure 4A). And in the IPITT, the plasma glucose levels in the 0.24% FRB group decreased promptly after the insulin administration (Figure 4E).

**Table 4:** Effect of fenugreek on the body weight, energy intake, tissue weights and plasma parameters of experiment 2

	**Ctrl**			**Fen**			**FRB**^**6**^								
							**0.12%**			**0.24%**			**1.2%**		
Total energy intake (kJ)	34,686	±	363	35,076	±	184	35,332	±	42*	35,119	±	196*	35,319	±	40
Final body weight (g)	757	±	21^ab^	792	±	13^a^	789	±	18^ab^	773	±	16^ab^	717	±	14^b^
EER^1^ (mg/kJ)	1.54	±	0.32	2.86	±	0.31	2.51	±	0.46	2.38	±	0.21	0.39	±	0.52*
Liver weight (g)	21.3	±	0.9	21.3	±	1.1	21.6	±	0.7	21.8	±	1.4	19.3	±	0.6
EWAT^2^ weight (g)	21.0	±	0.6	23.6	±	1.4	22.7	±	1.6	20.3	±	1.2	15.7	±	1.5*
Soleuse weight (g)	0.40	±	0.02	0.44	±	0.02	0.51	±	0.08	0.51	±	0.07	0.54	±	0.06*
Plasma TG^3^ (mmol/L)	1.34	±	0.24	1.73	±	0.36	1.49	±	0.34	1.43	±	0.37	1.27	±	0.26
Plasma TC^4^ (mmol/L)	1.95	±	0.11	1.80	±	0.05	1.81	±	0.12	1.76	±	0.15	1.60	±	0.09*
AST (U/L)	58.8	±	4.1	56.5	±	3.5	52.0	±	2.9	57.0	±	4.5	52.4	±	1.4
ALT (U/L)	19.6	±	2.0	21.1	±	2.5	18.3	±	1.8	20.3	±	3.5	25.0	±	4.0
AST/ALT^5^	1.29	±	0.18	1.13	±	0.07	1.18	±	0.08	1.29	±	0.21	0.95	±	0.11

Ctrl: Control group, Fen: Fenugreek group. ^1^EER (mg/kJ) = Body weight gain (mg)/Total food intake (kJ). ^2^EWAT: Epididymal white adipose tissue. ^3^TG: Triglyceride. ^4^TC: Total cholesterol. ^5^The relative values for Normal rats' data; AST/ALT = AST (U/L)/ALT (U/L)/Normal rats' data. ^6^FRB contains 96% of protodioscin converted to dioscin. Values are expressed as Mean ± SEM for 7–8 rats. Mean without a common superscript letter is significantly different, *P* < 0.05 (ANOVA). Asterisks indicate a difference from Ctrl: *P* < 0.05 (*t*-test).

**Table 5:** The insulin resistance index of experiment 2

	**Ctrl**			**Fen**			**FRB**^**4**^								
							**0.12%**					**0.24%**			**1.2%**		
FBG^1^ (mmol/L)	5.70	±	0.24	5.93	±	0.40	5.69	±	0.30	5.21	±	0.22	4.95	±	0.25*
FPI^2^ (nmol/L)	0.178	±	0.045	0.235	±	0.052	0.176	±	0.029	0.368	±	0.095	0.165	±	0.018
HOMA-IR^3^	1.00	±	0.24	1.47	±	0.39	0.97	±	0.12	1.88	±	0.51*	0.81	±	0.08

Ctrl: Control group, Fen: Fenugreek group. ^1^FBG: Fasting blood glucose. ^2^FPI: Fasting plasma insulin. ^3^Relative values of (fasting blood glucose × fasting blood insulin) to the mean value of Ctrl group. ^4^FRB contains 96% of protodioscin converted to dioscin. Values are expressed as Mean ± SEM for 7–8 rats. Mean without a common superscript letter is significantly different, *P* < 0.05 (ANOVA). Asterisks indicate a difference from Ctrl: *P* < 0.05 (*T*-test).

## Discussion

Our previous study showed that the effective dose of fenugreek was around 2.4% (w/w) [12]. The present study has shown that the effective dose of FRB is lower than that of fenugreek.

The previous study demonstrated that fiber sources providing predominantly IDF, such as corn bran, cellulose and wheat bran, have little effect on plasma and liver cholesterol levels. On the other hand, fiber sources containing a mixture of both SDF and IDF, such as soybean and oat bran, have intermediate effects on plasma and liver cholesterol levels [19]. SDF may be fermentable in the large intestine by the action of colonic bacteria producing short chain fatty acids such as butyrate, propionate, acetate etc. [20], which can act to reduce cholesterol synthesis [21]. And the major functions of IDF fractions can be attributed to their passive water holding capacity and non-digestibility, which may help to increase the bulk and shortens the transit time of stool through the intestinal tract [22]. However, IDF showed no effect on cholesterol absorption. The increase of SDF fraction in FRB might induce remarkable lowering effects on plasma and liver cholesterol concentrations [23].

On the other hand, many reports have been published, and demonstrated that diosgenin (aglycon of protodioscin and dioscin) had effects on glucose and lipid metabolism. Diosgenin raises the peroxisome proliferator-activated receptor γ (PPARγ) level in WAT, and promotes adipocyte differentiation and size reduction of adipocytes. As a result, the secretion of monocyte chemoattractant protein-1 (MCP-1) in adipocytes is suppressed, whereas the secretion of adiponectin is promoted, and the inflammation in adipose tissue is inhibited [13,24]. Therefore, diosgenin is also a candidate component to be contained in fenugreek for the improvement of insulin sensitivity. In addition, saponins, such as diosgenin, form large micelles from bile acid and saponin molecules in the small intestine, and these micelles inhibit the absorption of cholesterol by directly excreting it in feces [25,26]. Diosgenin also reduces the triglyceride content and mRNA expression levels of lipogenic genes (FAS, SCD-1 and ACC) and suppresses the LXRα transactivation, leading to the down-regulation of both the mRNA and protein expression levels of SREBP-1c in HepG2 cells [27]. Thus, diosgenin is considered to be an active ingredient. However, a major part of protodioscin was converted by the enzyme into dioscin in the FRB. Therefore, in our study, dioscin as well as diosgenin may have contributed to the improvement of glucose and lipid metabolism.

There was a discrepancy of plasma triglyceride levels between the two experiments. We suspect that the longer period of fasting before the sacrifice in the experiment 2 might have induced the low levels of plasma triglyceride.

The results of the experiment 1 and 2 suggested that FRB had effect on lipid metabolism and glucose tolerance in the 1.2%, 2.4% and 4.8% FRB groups. In the experiment 1, appetite declined significantly in the 2.4% and 4.8% FRB groups. And the soleus muscle weight and urinary creatinine levels (data not shown), which were related to the muscle mass as well as EWAT weight, decreased in these two groups. In addition, the plasma albumin and protein levels decreased in the 4.8% FRB group as compared with the Ctrl group (data not shown). Furthermore, the plasma insulin levels were relatively high, which led to the very low level of blood glucose at 120 min in the 4.8% FRB group. These results suggested that overdose of FRB induced a body and muscle weight reduction, and caused a dangerous hypoglycemia.

We have demonstrated that 1.2% or more of FRB prevents diet-induced metabolic disorders including insulin resistance, dyslipidemia and fatty liver. The effective and safe dose of FRB is around 1.2% (w/w) contained in the diet (~ 0.25 g/kg BW/day).

## Abbreviations

FRB, Fenugreek with reduced bitterness; HFS, High-fat high-sucrose; EWAT, Epididymal white adipose tissue; 4-OH-Ile, 4-Hydroxyisoleucine; OGTT, Oral glucose tolerance test; IPITT, Intraperitoneal insulin tolerance test; AST, Aspartate amino transferase; ALT, Alanine transaminase; EER, Energy efficiency ratio; HOMA-IR, Homeostasis model assessment as an index of insulin resistance; AUC, Area under the curve; SDF, Water-soluble dietary fiber; IDF, Insoluble dietary fiber; PPARγ, Peroxisome proliferator-activated receptor γ; MCP-1, Monocyte chemoattractant protein-1.

## Competing interests

The authors declare that they have no competing interests.

## Authors' contributions

EM conceived the study and its design, and wrote the manuscript, carried out the experiments, and analyzed data. HC participated in the study design, assisted to carry out the experiments and draft the manuscript. KT and SH prepared fenugreek and FRB, and participated in the study design. NT and NT participated in the study design and assisted to draft the manuscript. KK conceived the study, participated in its design and helped to draft the manuscript. All authors read and approved the final manuscript.

## Authors’ information

^1^Department of Clinical Dietetics & Human Nutrition, Faculty of Pharmaceutical Sciences, Josai University, Saitama 350–0295, Japan.

^2^House Foods Corporation, Somatech Center, Chiba 284–0033, Japan.

## Supplementary Material

Additional file 1 **The English version of reference 13.** Reference 13 derives from the abstract in Japanese of the original presentation. Here we translated it into English. Click here for file
