# Supplementary material for: Fenugreek with reduced bitterness prevents diet-induced metabolic disorders in rats
Source: Lipids Health Dis. 2012 May 29;11:58. doi: 10.1186/1476-511X-11-58 (PMC3416582; doi:10.1186/1476-511X-11-58)
Supplement: Additional file 1 — The English version of reference 13. Reference 13 derives from the abstract in Japanese of the original presentation. Here we translated it into English. [file 1476-511X-11-58-S1.pdf]

Abstracts of the 14th Annual Meeting of Japan Society for Spice Research

Nihon Kōshiryō Kenkyūkai kōen yōshishū

November 13 (Saturday), 1999

University Hall, Sugiyama Jogakuen University

Regarding bitter components in fenugreek (*Trigonella foenum-graecum* L.) seeds

House Foods Corporation (Somatech Center): Masamura, N. and Sawada, H.

[Objective]

A pulverized product of fenugreek ("*Methi*" in Hindi; "*Koroha*" in Japanese) seeds is a spice which is mixed with a curry powder and contributes to the generation of curry flavor. However, meanwhile, fenugreek seeds have distinctive strong bitter taste. Thus, it is difficult to use fenugreek seeds in large amounts.

We are conducting research in order to suppress the bitter taste of fenugreek while maintaining the original flavor of fenugreek. For the first step of research, we identified bitter components in fenugreek seeds. The results are reported below.

[Method and Results]

A pulverized product of fenugreek seeds was used as a material. The product was purified by solvent extraction and column chromatography shown in the next page with the confirmation of the presence or absence of bitter taste. Accordingly, bitter taste components were isolated. In addition, each extract or fraction was concentrated and dried. Then, each resultant was resuspended and dissolved in a given amount of water for bitter taste evaluation.

The obtained diethyl ether extract of the pulverized product of fenugreek seeds was found to have no bitter taste. Next, the pulverized product of fenugreek seeds subjected to ether extraction was further subjected to methanol extraction. The resulting extract was found to have strong bitter taste upon sensory evaluation. Meanwhile, a residue obtained after methanol extraction was found to have no bitter taste. Accordingly, it was judged that all bitter components in fenugreek seeds can be extracted with methanol.

Polysaccharides contained in large amounts in a methanol extract of the pulverized product of the seeds inhibited separation in the subsequent purification step. Therefore, the methanol extract was resuspended in a small amount of water for ethanol precipitation. Bitter components were not precipitated by ethanol precipitation treatment, resulting in the separation of the components from precipitated polysaccharides. The supernatant obtained after ethanol precipitation treatment was subjected to butanol extraction. As a result, only the butanol layer was found to have bitter taste.

The butanol extract was fractionated by ODP MPLC (stepwise elution (mobile phase: ethanol:water = 30:70, 40:60, or 50:50)). Each eluted fraction was subjected to sensory evaluation. Only one fraction (ethanol:water = 40:60) was found to have bitter taste. The fraction was further subjected to separation by ODS HPLC (mobile phase: acetonitrile:water = 25:75). A fraction of each peak was obtained and subjected to sensory evaluation. As a result, some peak components were found to have bitter taste.

Among the bitter components isolated above, the component obtained in the largest amount was subjected to structural analysis by NMR and the like. As a result, the component was confirmed as protodioscin, which is a furostanol saponin and has been reported to be isolated from fenugreek seeds. The other bitter components were also assumed to be furostanol saponins because they developed red color when stained with an Ehrlich reagent.

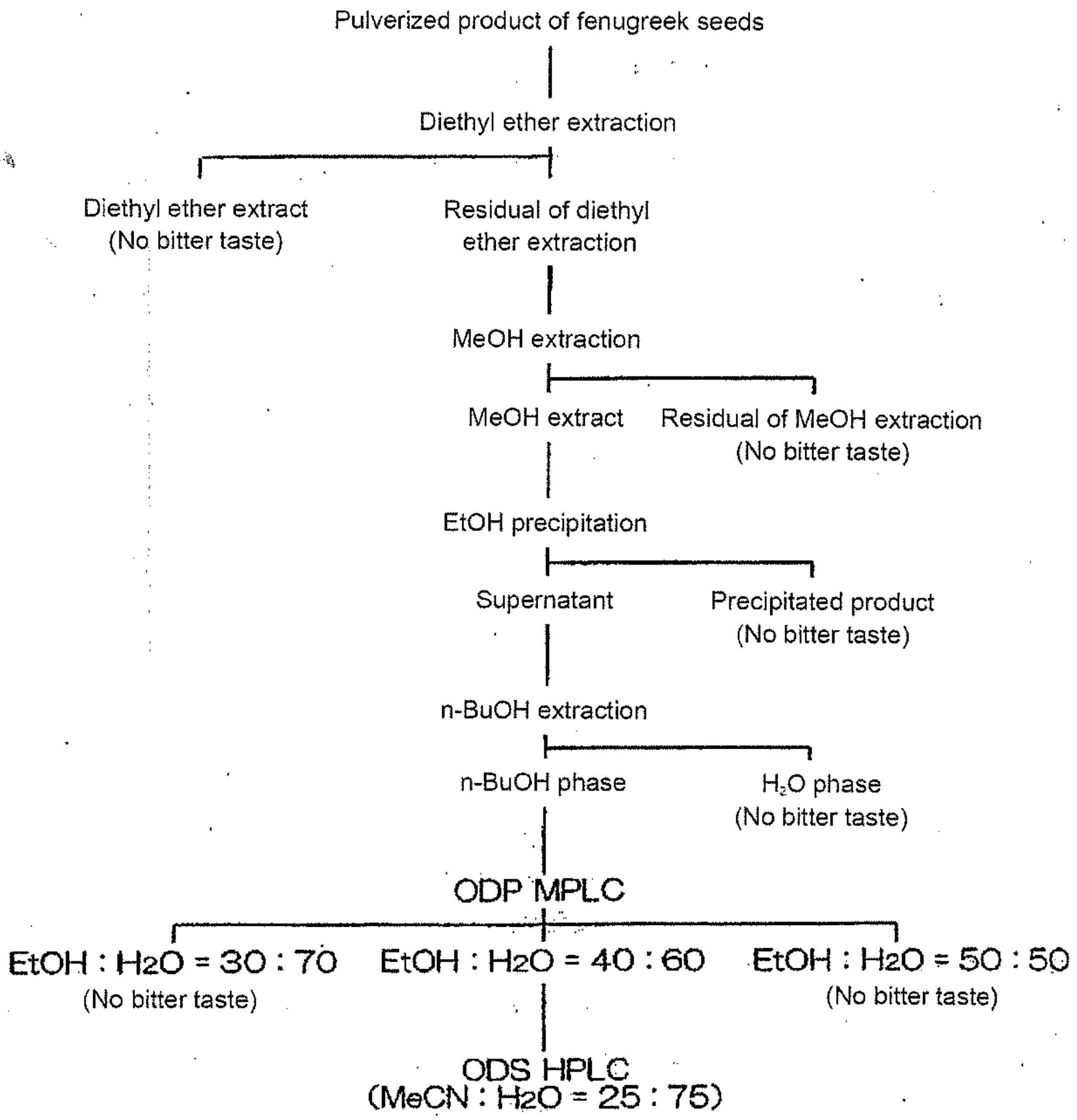

Scheme of purification of bitter components from pulverized product of fenugreek seeds
